# Quality of life in elderly people after a hip fracture: a prospective study

**DOI:** 10.1186/s12955-020-01314-2

**Published:** 2020-03-14

**Authors:** Francisco Javier Amarilla-Donoso, Fidel López-Espuela, Raúl Roncero-Martín, Olga Leal-Hernandez, Luis Manuel Puerto-Parejo, Ignacio Aliaga-Vera, Rosaura Toribio-Felipe, Jesús María Lavado-García

**Affiliations:** 1 Department of Nursing, Hospital Campo Arañuelo de Navalmoral de la Mata, Cáceres, Spain; 2 Nursing Department, Nursing and Occupational Therapy College, University of Extremadura, Avda. De la Universidad S/N. CP: 10003, Caceres, Spain; 3 Department of Stomatology II, University of Madrid, Complutense, Madrid, Spain; 4 Department of Nursing, Hospital Virgen del Puerto, Plasencia, Plasencia, Spain

**Keywords:** Geriatric care, Hip fracture, Health-related quality of life

## Abstract

**Background:**

Hip fracture is an important social and medical problem due to its increasing prevalence, the consequences for health and the economic impact on the health care system, but there is no doubt that it also has repercussions on health-related quality of life (HRQoL). Hence the importance of understanding and determining the impact of the condition on everyday life from the perspective of the patient’s physical, emotional and social well-being.

**Purpose:**

To determine the impact of hip fracture on HRQoL of people over the age of 65 1 month after surgery, related factors and the effects on functional ability and mood.

**Methods:**

Prospective observational study conducted in the traumatology units of two university hospitals in the province of Cáceres with consecutive sampling of all patients over the age of 65 admitted for hip fracture surgery during the study period. Sociodemographic and clinical data were recorded at the time of admission and prospectively at the follow-up visit 1 month later. Clinical, social, quality of life (EQ-5D-), basic functional and instrumental capacity (Barthel Index (BI) and Lawton & Brody Scale), and geriatric depression (Yesavage) variables were collected.

**Results:**

The study included 224 patients with a median age of 84.6 years (SD ± 6.1), 76.3% were female. Charlson’s comorbidity was 5.3 (SD ± 1.2). The EQ-5D index decreased from 0.62 (SD ± 0.35) to 0.16 at 1 month follow up (SD ± 0.20) *p* <  0.001. The mean Visual Analog Scale (VAS) score of EQ-5D decreased from 72.8 (SD ±15.8) to 48.3 (SD ± 17.2) p <  0.001. All dimensions of EQ-5D showed a significant reduction from the time of pre-fracture status to 1 month after surgery. Independent factors associated with HRQoL 1 month after surgery were pre-fracture status Barthel Index score, Lawton and Brody scale, presence of depression, and type of surgery.

**Conclusions:**

After a hip fracture, patients experience considerable deterioration in their HRQoL, especially in self-care, daily activities, and mobility. There is also a significant decline in functional capacity for both the basic and instrumental activities of daily living. One month after surgery, HRQoL is a long way from pre-fracture levels.

## Introduction

Hip fracture is an important social and medical problem due to its increasing prevalence, the consequences for health and the economic impact on the health care system [1]. It is estimated that approximately 347,564 hip fractures will occur in Spain in the decade from 2015 to 2025. In 2017 there were 73,381 hip fracture discharges, 71% of whom were women [2]. According to the World Health Organization, the number of hip fractures associated with osteoporosis will triple over the next 50 years, from 1.7 million cases in 1990 to 6.3 million in 2050 worldwide [3]. Approximately one-third of women and one in twelve men will suffer a hip fracture in their lives [4]. Age, osteoporosis, and falls are major risk factors for hip fractures [1]. More than 85% of hip fractures occur in people older than 65 [5]. Age-related decline characterized by reduced neuromuscular coordination, vision, balance, and reaction time are associated with falls and hip fractures resulting in fragile elders [6, 7]. These events lead to a deterioration of strength and pace.

Fractures cause significant impairment of the ability to independently perform basic daily activities, such as mobility (especially climbing stairs), dressing or bathing [8]. Between 25 and 75% of people who walked independently before the fracture become dependent after 1 year, or do not reach the same pre-fracture level of autonomy [9]. Hip fracture has also been associated with higher co-morbidity and mortality rates. One-year hip fracture mortality ranges from 18 to 33% [10] and the patients with hip fractures have five to eight times greater mortality than patients without fractures within 3 months of their events, with the increased risk persisting even after 10 years [11]. The estimated average direct cost of caring for a hip fracture in Spain is €8400, with global figures ranging between €300–860 million [12].

There are countless articles and reports that evaluate the impact of hip fracture in quantitative terms (mortality, morbidity, life expectancy) and cost, but fewer that consider qualitative indicators that express the impact on quality of life, patient satisfaction and associated factors [13–15]. The importance of assessing health-related quality of life (HRQoL) stems from the need to understand and determine the impact of the condition on daily life by focusing on the patient’s physical, emotional, and social well-being. In addition, it provides information on the effectiveness of therapeutic treatments without overlooking the fact that state of health is profoundly influenced by mood, coping mechanisms, social support, socioeconomic conditions and health care services, with important repercussions on health-related quality of life outcomes (Alexiou, 2018). These aspects of vital importance to human life will clearly have the most influence on the patient’s evolution. Thus, factors that can interfere with the patient’s HRQoL include the type of treatment, delayed surgery and the emergence of post-surgical complications [16]. Similarly, family and social support are thought to influence perceived quality of life. Upon discharge, patients may go home, be cared for (or not) by family members/caregivers, or be institutionalised (in cases of significant functional impairment or lack of a social support network).

There is scarce information on the factors that influence HRQoL in the short term in the Spanish population, once the immediate post-operative period is over. Therefore, the purpose of this study was to determine the impact of hip fracture on the HRQoL of people affected by a hip fracture 1 month after surgery as well as the conditioning factors.

The secondary purpose of the study was to identify the effect of fracture stabilization on functional capacity, mood and socio-familial situation.

## Design methods

Prospective observational study conducted in the traumatology units of two university hospitals in the province of Cáceres between June 2015 and June 2016.

Consecutive sampling was done. The inclusion criteria were patients over the age of 65 admitted with a primary diagnosis of hip fracture who underwent emergency surgery for surgical reduction of the fracture; patients without cognitive impairment and who were not terminally ill; and those without linguistic barriers that would prohibit them from understanding the questionnaires.

All patients included in the study were treated according to standard clinical practice. Written informed consents were required to participate in the study. The procedures were compliant with the Declaration of Helsinki and were approved by the Clinical Research Ethics Committee of Cáceres (Spain).

### Data collection

A questionnaire containing the following variables was drawn up for data collection: clinical, socio-demographic and economic data, personal history, standard treatment, clinical variables of functional dependence (Barthel Index, Lawton-Brody Scale), and social-familial assessment (Gijón Assessment Scale), health-related quality of life variables (EuroQol-5D), days of hospitalisation, delay in surgical intervention, destination after discharge, functional ambulation capacity, presence of complications and polymedication. The Charlson Comorbidity Index (CCI) was used as a method to quantify the number of chronic disorders and their severity. The American Society of Anesthesiologists (ASA) scale was used to classify physical fitness. The questionnaire was completed face to face at the time of admission and 1 month after discharge from hospital during the follow-up visit. Data were obtained through personal interviews with patients and reviews of hospital medical records. To ascertain the pre-fracture status, participants were interviewed about their functional status presence of symptoms of depression, social situation and quality of life 2 weeks before the fracture and, this information being identified as the baseline condition, or pre-fracture status.

HRQoL was assessed using the EuroQol-5D-3 L questionnaire [17]. The EuroQol-5D is a descriptive system with five domains (mobility, self-care, regular activities, pain/discomfort and anxiety/depression) divided into three levels of severity: no problems, some problems, extreme problems (labelled 1–3; where 1 indicates that there is no problem, 2 some problems, and 3 extreme problem), from which a weighted score is derived based on cultural and national differences. It also includes a visual analogue scale (EQ-5D VAS) [18] defined by a 20 cm vertical scale at either end of which are the extreme expressions of self-perceived state of health ranging from 0 (worst health) to 100 (best health). Responses to the state of health classification system were converted to an overall score using a published algorithm for the Spanish population [19].

The ability to perform basic activities of daily living (BADL) was assessed using the Barthel Index [20]. This scale evaluates ten elements (feeding, bathing, grooming, dressing, bowels, bladder, toilet use, transfers, mobility and stairs). A total score between 0 and 20 suggests total dependence for the performance of BADL; 21 to 60, severe dependence; 61 to 90, moderate dependence; 91 to 99, mild dependence; and 100, independence [21]. The ability to perform instrumental activities of daily living (IADL) was assessed using the Lawton and Brody scale [22], which assesses eight items (ability to use the telephone, shopping, food preparation, housekeeping, laundry, mode of transportation, responsibility for own medications and ability to handle finances). Taking gender differences into account, total dependency was categorised as 0 in men and 0–1 in women; severe as 1 in men, 2–3 in women; moderate as 2–3 in men and 4–5 in women; minor as 4 in men and 6–7 in women; and independent as 5 in men and 8 in women. Ambulation capacity was determined through the use of functional ambulation categories [23], a scale with 6 possible scores (0–5), where the lower the score, the greater the dependence. A total score of 0–3 indicated that the patient was dependent or non-ambulatory; 4–5 suggested independence. Symptoms of depression in geriatric patients were detected using the Spanish version of the 15-point Yesavage Depression Scale [24, 25]. A score of 0–5 indicated no depression; 6–9 suggested possible depression, and ≥ 10 revealed an established depression. The socio-familial situation was determined by the Gijón Socio-familial Scale [26] which assesses 5 dimensions (family situation, economic situation, housing, social relations and social support network). A total score between 5 and 9 indicates a good or adequate social situation; 10–14 indicates social risk, and ≥ 15 indicates a social problem.

Comorbidity was calculated using the Charlson Comorbidity Index (CCI) [27]. This is a predictive model which assigns numerical values to different chronic pathologies, obtaining the final score for each individual patient by adding the partial values. We identified patients who took 5 or more medications daily for a period of more than 6 months as polymedicated.

### Statistical analysis

For the descriptive analysis, we calculated percentages for the categorical variables and the mean with standard deviation (SD) for the distribution of the continuous variables: age, EQ-5D VAS, EQ-5D Index, the Charlson Comorbidity Index, the Barthel Index, the Yasavage Depression Scale and the Gijón Index.

Consistency with normal distribution was checked with the Kolgomorov-Smirnov test and the Student’s t-test or Mann-Whitney U-test for quantitative variables, respectively, depending on whether or not they followed that distribution and the c^2^ or Fisher’s exact test for categorical variables, depending on the case. The relationship between quantitative variables was analysed using the Spearman’s Rho correlation coefficient.

Paired groups (pre-fracture status/one-month comparison) were analysed using the Wilcoxon signed-rank test for the continuous variables (Barthel Index, Gijón Scale, Yasavage Scale and EQ-5D VAS) and the McNemar test for the categorical variables (institutionalisation and discharge date) and Cochran’s Q Test for EQ-5D quality of life dimensions.

Considering HRQoL as a dependent variable, measured with the EQ-5D index and the EQ-5D VAS, the relationship between HRQoL and the independent variables was analysed in order to identify the factors related to it.

A multiple linear regression model was constructed to find the independent variables associated with the HRQoL at 1 month. We included variables whose significance had been *p* <  0.05 in the univariate analysis, using the stepwise regression method to adjust the model with all initially introduced variables, eliminating the independent variables that present collinearity from the analysis and showing the regression coefficients (B) and their respective 95% confidence intervals (95% CI). The significance level for the different analyses was established as *p* <  0.05. The data analysis was performed using SPSS Statistics for Windows, version 20.

## Results

A total of 270 patients were admitted for hip fracture during the study period. Of these, 3 (1.1%) refused to participate in the study, 43 (15.92%) met one of the exclusion criteria, and 2 (0.8%) died before they could be captured for inclusion in the study. A total of 224 patients were included in the study. Mortality during the first post-discharge month was 5 patients (2.2%).

The socio-demographic and clinical characteristics of the patients are shown in Table 1. The mean age of the study participants was 84.6 years (SD ± 6.1 years); the majority of patients were women (76.3%) and polymedicated (69.6%). 64.3% of the patients suffered a trochanteric fracture, compared to 35.7% who suffered a neck fracture. Fracture reduction by intramedullary rod was the most common type of surgical procedure (66.2%). Most surgeries were performed under spinal anaesthesia (79.5%). The time between hospital admission and surgery was 3.0 days (SD ± 2.8 days) and the hospital stay was 5.3 days (SD ± 1.2 days). Charlson’s Comorbidity Index at the time of surgery was 5.3 (SD ± 1.2).

**Table 1:** Sociodemographic and clinical characteristics of patients at hospital admission

Variables	Total patients (*n* = 224)
Sex female, n (%)	171 (76.3)
Age; mean years ± *SD*	84.6 (± 6.1)
Age, Groups, n (%)	
< 85 years	106 (47.3)
≥ 85 years	118 (52.7)
Study level, n (%)	
No studies	85 (37.9)
Primary	130 (58)
Secondary	6 (2.7)
University	3 (1.3)
Living status, n (%)	
Living alone	57 (25.4)
Living in couple	61 (27.2)
Living with relatives	62 (27.7)
Supervised flat	7 (3.1)
Residency	37 (16.5)
Clinical history, n (%)	
Osteoporosis	21 (9.4)
Previous hip fracture, n (%)	18 (8.0)
Polymedicated, n (%)	156 (69.6)
Charlson comorbidity index, mean (SD)	5.3 (±1.2)
Type of fracture, n (%)	
Neck	80 (35.7)
Trochanter	144 (64.3)
Type of surgical intervention, n (%)	
Intramedullary nail	147 (65.6)
Hip replacement (prosthesis)	75 (33.5)
Other surgical treatments	2 (0.9)
Complications during the surgery, n (%)	33 (14.71)
ASA PS for peri-operative risk, n (%)	
I	1 (0.4)
II	70 (31.3)
III	136 (60.7)
IV	17 (7.6)
Type of anesthesia (%)	
General	2 (0.9)
Spine	178 (79.5)
Spine with sedation	44 (19.6)
Previous hip fracture, n (%)	18 (8.0)
Time elapsed between hospital admission and intervention, mean (SD)	3.02 (± 2.8)
Time elapsed between hospital admission and discharge, mean (SD)	5.3 (±1.2)

*ASA PS* American Society of Anesthesiologists physical status, *SD* Standard Deviation

Changes in the socio-demographic and clinical characteristics of patients before surgery and 1 month after surgery are shown in Table 2. 80.4% of the patients lived at home prior to the fracture. The percentage of institutionalised patients increased significantly from pre-fracture status (19.6%) to 1 month after surgery (41.1%), *p* <  0.001.

**Table 2:** Change in sociodemographic and clinical characteristics in patients between pre-fracture status and after one month of the hip fracture

Variables	Hospital admission (pre-fracture status) *N = 224*	After one month of surgery *N = 219*	*p* with pre-fracture
EQ-5D Index, mean (SD)	0.62 (± 0.35)	0.16 (±0.20)	< 0.001
EQ-5D VAS, mean (SD)^a^	72.7 (±15.8)	48.3 (±17.2)	< 0.001
Living status, n (%)			
Non-institutionalized	180 (80.4)	129 (58.9)	< 0.001
Institutionalized	44 (19.6)	95 (41.1)	< 0.001
Barthel index, mean (SD)^a^	87.5 (±16.8)	53.5 (±17.7)	< 0.001
IADL of Lawton and Brody, mean (SD)^a^	5.05 (±2.6)	2.15 (±1.4)	< 0.001
Functional Ambulation Classification (FAC): Do not walk independently, n (%)^b^	95 (42.8)	217 (99.1)	< 0.001
Gijon scale, mean (SD)^a^	8.3 (±1.9)	8.4 (±1.9)	0.090
Yesavage Depression Scale, mean (SD)^a^	3.2 (±3.6)	5.6 (±3.2)	< 0.001

^a^test Wilcoxon

^b^test McNemar

*IADL* Instrumental activities of daily living scale, *SD* Standard deviation

Regarding quality of life, mean index scores for EQ-5D and EQ-5D VAS significantly decreased 1 month after surgery, from 0.62 (SD ± 0.35) corresponding to the pre-fracture status, to 0.16 (SD ± 0.20) (*p* <  0.001) and 72.7 (SD, ±15.8) to 48.3 (SD, ±17.2) p <  0.001, respectively. Changes in the dimensions of the EQ-5D questionnaire between the pre-fracture situation and 1 month after surgery are shown in Table 3. All domains were significantly affected. Compared to pre-fracture status, the proportion of patients reporting problems at 1 month more than doubled in the self-care dimension (55%), nearly doubled in activities of daily living (44%) and mobility (41%), and although significant differences were evident in the dimensions of pain (27%) and anxiety/depression (16%), the increase was more discreet. Moreover, a significant increase was seen in patients reporting extreme problems related to self-care (from 7.1 to 44.3%; *p* <  0.001) and usual activities (from 22.3 to 81.3%; *p* <  0.001) 1 month after surgery.

**Table 3:** Changes in dimensions from the eq-5d questionnaire through the follow-up period, and change in the functional dependence level and depression in patients between hospital admission and after one month of the hip fracture

	Pre-fracture n (%)	1 month n (%)	*p* with pre-fracture ^a^
EQ-5D questionnaire			
Mobility			< 0.001
No problems	96 (42.9)	1 (0.5)	
Some problems	127 (56.7)	215 (98.2)	
Severe problems	1 (0.4)	3 (1.4)	
Self-care			< 0.001
No problems	134 (59.8)	4 (1.8)	
Some problems	74 (33.0)	118 (53.9)	
Severe problems	16 (7.1)	97 (44.3)	
Usual activities			< 0.001
No problems	107 (47.8)	2 (0.9)	
Some problems	67 (29.9)	39 (17.8)	
Severe problems	50 (22.3)	178 (81.3)	
Pain/discomfort			< 0.001
No problems	98 (43.8)	32 (14.6)	
Some problems	104 (46.4)	177 (80.8)	
Severe problems	22 (9.8)	10 (4.6)	
Anxiety/depression			< 0.001
No problems	145 (64.7)	103 (47.0)	
Some problems	71 (31.7)	103 (47.0)	
Severe problems	8 (3.6)	13 (5.9)	
Barthel Index			
Total dependent		9 (4.1)	< 0.001
Severe dependent	4 (1.8)	24 (11.0)	
Moderate dependent	14 (6.3)	100 (45.7)	
Mild dependent	105 (46.9)	84 (38.4)	
Independent	101 (45.1)	2 (0.9)	
Lawton and Brody Index			
Total dependent	24 (10.7)	65 (29.7)	< 0.001
Severe dependent	33 (14.7)	106 (48.4)	
Moderate dependent	49 (21.9)	40 (18.3)	
Mild dependent	37 (16.5)	5 (2.3)	
Independent	81 (36.2)	3 (1.4)	
Geriatric Depression Scale			
Normal	170 (75.9)	127 (58)	< 0.001
Depression probably	35 (15.6)	59 (26.9)	
Established depression	19 (8.5)	33 (15.1)	

^a^Cochran

Table 3 shows the changes observed in levels of functional dependence and mood. For functional capacity variables, there was a significant drop in both the Barthel and the Lawton and Brody scale scores (*p* <  0.001). In terms of mood, 75.9% had no depression in the pre-fracture assessment compared with 42% in patients presenting with established or probable depression 1 month after surgery (*p* <  0.001).

In the univariate analysis, (Tables 4 and 5) we found eight parameters that correlated significantly with the EQ-5D index at 1 month. These parameters included patient age, pre-fracture Charlson Comorbidity Index, pre-fracture Barthel Index, pre-fracture Yesavage Depression Scale, pre-fracture Lawton and Brody Scale, pre-fracture residential status, type of intervention, and FAC. The strongest correlations were found with the Barthel Index (Spearman’s Rho coefficient = 0.578), Lawton and Brody (Spearman’s Rho coefficient = 0.541) and Yesavage (Spearman’s Rho coefficient = − 0.317).

**Table 4:** Factors associated with index eq-5dand eq-5dvas after one month of the surgical intervention

	EQ-5D Index Month (n = 219)		Difference EQ-5DIndex Month -pre-fracture		EQ-5D VAS 1 Month (n = 219)		Difference EQ-5D VAS Month -pre-fracture	
	Rho^a^	*p*-value	Rho^a^	*p*-value	Rho^a^	*p*-value	Rho^a^	*p*-value
Age	−0.252	0	− 0.139	0.039	− 0.115	0.089	− 0.077	0.258
Time elapsed between hospital admission and intervention	0.045	0.09	0.048	0.476	0.045	0.506	−0.015	0.824
Time elapsed between hospital admission and discharge	0.05	0.463	0.048	0.476	0.09	0.182	−0.007	0.917
Charlson comorbidity index	−0.151	0.026	0.055	0.419	−0.124	0.069	−0.015	0.823
Pre-fracture Barthel Index	0.578	0	−0.04	0.556	0.352	0	−0.094	0.168
Pre-fracture Lawton y Brody Index	0.651	0	0.241	0	0.544	0		
Pre-fracture Yesavage Depression Scale	−0.317	0	0.223	0.001	−0.282	0	0.137	0.042
Pre-fracture Gijon scale (not institutionalized)	−0.131	0.081	−0.12	0.076	−0.045	0.556	−0.01	0.894

^a^*Spearman’s Rho correlation*

**Table 5:** Factors associated with index eq-5dand eq-5dvas after one month of the surgical intervention

		EQ-5DIndex 1 Month		Difference EQ-5DIndex 1 Month -pre-fracture		EQ-5D VAS 1 Month		Difference EQ-5DVAS Month - pre-fracture	
		Mean	*p*-value^a^	Mean	*p*-value^a^	Mean	*p*-value^a^	Mean	*p*-value^a^
Sex	Male	−0.07 (0.43)	0.39	−0.74 (0.33)	0.113	46.3 (19.2)	0.32	−28.9 (18.9)	0. 107
	Female	−0.04 (0.48)		−0.64 (0.38)		48.5 (16.3)		−23.3 (17.6)	
Living status	Non-institutionalized	−0.02 (0.46)	0.026	−0.67 (0.36)	0.742	48.9 (17.7)	0.215	−25 (18)	0.433
	Institutionalized	−0.19 (0.5)		−0.62 (0.42)		44.5 (13.7)		−23 (17.8)	
Polymedicated	No	0 (0.45)	0.296	−0.74 (0.26)	0.287	52.4 (19.6)	0.016	−26.5 (18.6)	0.553
	Yes	−0.07 (0.48)		−0.63 (0.41)		46.2 (15.5)		−23.8 (17.7)	
Type of surgical intervention	Intramedullary nail	−0.1 (0.46)	0.024	−0.71 (0.34)	0.01	47.3 (16.6)	0.257	−24.3 (16.8)	0.685
	Hip replacement (prosthesis)	0.04 (0.48)		−0.57 (0.42)		49.4 (17.8)		−25.2 (20.2)	
Complications	No	−0.05 (0.48)	0.732	−0.67 (0.36)	0.781	47.5 (17.6)	0.06	−25.2 (17.3)	0.432
	Yes	−0.08 (0.44)		0.61 (0.44)		51.7 (12.6)		−20.3 (21.8)	
Type of fracture	Neck	0.03 (0.49)	0.053	−0.58 (0.42)	0.036	48.3 (18)	0.664	−25.7 (20)	0.43
	Trochanter	−0.09 (0.46)		−0.71 (0.34)		47.9 (16.5)		−24 (16.8)	
Previous hip fracture	No	−0.04 (0.47)	0.309	−0.66 (0.37)	0.78	48.1 (17.1)	0.877	−24.3 (17.9)	0.743
	Yes	−0.13 (0.45)		−0.66 (0.4)		47.8 (16.6)		−27.2 (18.7)	
Functional Ambulation Classification (FAC)	Do not walk independently	−0.3 (0.5)	0	−0.67 (0.44)	0.019	43.5 (13.9)	0	−22.7 (16.5)	0.117
	Walk independently	0.13 (0.35)		−0.66 (0.31)		51.5 (18.4)		−26 (19)	

^a^U Mann-Whitney

Six parameters (patient age, pre-fracture Barthel Index, pre-fracture, Yesavage Depression Scale, type of intervention, fracture type and FAC) correlated significantly with the difference between the pre-fracture EQ-5D index and the EQ-5D index 1 month after surgery. Correlation coefficients were lower compared to coefficients between the EQ-5D index and patient characteristics (Tables 4 and 5).

The strongest correlation between EQ-5D VAS score at 1 month was with Lawton and Brody (r = 0.401), the pre-fracture Barthel Index (r = 0.352), and the Yesavage Depression Scale (r = − 0.282) (Tables 4 and 5).

In the multiple regression analysis (adjusted R2 = 0.446), the Yesavage Depression Scale (B = -0.021 (− 0.035;-0.008), *p* <  0.002) was significantly associated with a lower EQ-5D index 1 month after surgery, while the Barthel Index (B = 0.012 (0.008;0.016), *p* <  0.001) and Lawton Brody pre-fracture (B = 0.040 (0.015;0.064), *p* <  0.001), were associated with higher EQ-5D index levels (Table 6).

**Table 6:** Multiple regression analysis of factors influencing eq-5d index at one month

EQ-5D Index	B	β	Confidence Interval of 95,0% to B	*P* value	R2 ajusted
Constant	−1.298		(−1.592; −1.003)	< 0.001	0.446
Pre-fracture Barthel Index	0.012	0.429	(0.008; 0.016)	< 0.001	
Pre-fracture Lawton y Brody Index	0.040	0.226	(0.015; 0.064)	0.001	
Pre-fracture Yesavage Depression Scale	−0.021	−0.166	(− 0.035; − 0.008)	0.002	
Constant	−1.145		(− 1.435; − 0.854)	< 0.001	0.045
Pre-fracture Barthel Index	0.004	0.159	(0.001; 0.007)	0.019	
Type of surgical intervention	0.124	0.158	(0.02; 0.228)	0.019	

B: Non-standardized regression coefficients

β: Standardized regression coefficients

As for the difference between the EQ-5D Index just before surgery and 1 month after surgery, the Barthel Index (B = 0.004 (0.001;0.007); *p* = 0.019) and the type of intervention (arthroplasty, B = 0.124 (0.02;0.228); *p* = 0.019) are identified as independent variables (adjusted R2 = 0.045) (Table 7). In the multiple regression analysis of EQ-5D VAS at 1 month (adjusted R2 = 0.184), a higher pre-fracture Lawton and Brody score was associated with a higher quality of life VAS score (B = 2.231 (1.44;3.022); *p* <  0.001=, while the Yesavage Depression Scale was associated with a lower 5D EQ VAS score (B = -0.931 (− 1.513;-0.35); *p* = 0.002) (Table 8).

**Table 7:** Multiple regression analysis of factors influencing difference between index prior to fracture and at one month

Difference EQ-5DIndex - pre-fracture −1 Month	B	β	Confidence Interval of 95,0% to B	*P* value
Constant	−1.145		(−1.435; −0.854)	< 0.001
Pre-fracture Barthel Index	0.004	0.159	(0.001; 0.007)	0.019
Type of surgical intervention	0.124	0.158	(0.02; 0.228)	0.019

B: Non-standardized regression coefficients

β: Standardized regression coefficients

R2 ajusted: 0,045

**Table 8:** Multiple regression analysis of factors influencing eq. 5d vas at one month

EQ-5D VAS	B	β	Confidence Interval of 95,0% to B	*P* value	R2 ajusted
Constant	37.593		(31.704; 43.482)	< 0.001	0.184
Pre-fracture Lawton y Brody Index	2.231	0.349	(1.44; 3.022)	< 0.001	
Pre-fracture status Yesavage Depression Scale	- 0.931	−0.198	(−1.513; − 0.35)	0.002	

B: Non-standardized regression coefficients

β: Standardized regression coefficients

## Discussion

Hip fractures are the most frequent cause of admission to trauma units in older people. There is broad consensus that surgery is the gold standard for the treatment of hip fractures, with the aim of regaining pre-facture functional ability to the extent possible.

There is little information on the factors that influence HRQoL in the short term in the Spanish population after the immediate post-operative period. The study conducted by [28] evaluated health-related quality of life in patients with subcapital femur fractures undergoing different haemostatic treatments, where the EQ-5D scale was used with five severity levels, while the Úbeda study [29] evaluated quality of life in patients with hip arthroplasty secondary to osteoarthritis, where fracture was an exclusion criterion.

Our study population is mainly composed of women over the age of 80, not institutionalized, which is in line with other studies [16, 28–33]. The length of hospitalisation was 5.3 days (±1.2), considerably less than what was reported in the Úbeda study.

Also noteworthy, as reported in other studies [14, 28, 34], the large impact of the domains of self-care, daily activities, mobility, pain or discomfort prior to the fracture. On the other hand, the pre-fracture EQ-5D VAS is around 73 (SD ± 15.8), higher than the data gathered by other studies [28, 32, 34], but similar to those found in the [35] study. This difference cannot be explained by the presence of comorbidity, since the values indicating the existence of pathology are higher in our study (CCI and ASA), nor by the greater functional deficiencies or worse mood, since the pre-fracture values of the Barthel Index and the depression scale are similar in our study (Barthel 87.5 ± 16.8; Depression scale 24.1% with established or probable depression) to those reported in the Buecking study [34]: Barthel 80 ± 25; Depression scale 24% with established or probable depression.

Comparing the pre-fracture EQ-5Dindex of 0.62 (SD ± 0.35) to the one-month post-fracture of 0.16 (SD ± 0.20), there is a considerable reduction, which is also evident in the EQ-5D VAS with a reduction of 24.4 points. The study of 4-week follow-up times [32] showed less variation between pre-fracture scores and 4 weeks out, 0.35 compared to 0.46 in our study. The study with a follow-up period through hospital discharge also showed less variation than that reflected in our study (0.71 (±0.29) - 0.21 (±0.46) [34].

The work carried out by [36] showed variations of 0.78 before the fracture, 0.59 at 4 months and 0.51 at 17 months. The smaller difference between the pre-fracture value and successive values compared to our study can be explained by the increase in the quality of life as a result of the recovery and rehabilitation process [33]. Another possible explanation for higher scores over longer follow-up periods could be the “response to change,” where patients become accustomed to their illness or experience changes in their expectations about their state of health [37].

The EQ-5D index scores could be expected to increase as recovery progresses [33], although few studies have examined the pattern of recovery after hip fracture, which is non-linear with higher gains in the first 1–2 months and slower recovery in later months [38]. However, extensive literature has shown that recovery is unlikely to restore pre-facture functional values.

Although most of our patients reported some sort of problem with all dimensions of EQ-5D 1 month after surgery, it is interesting to note that the areas identified as most affected were self-care and usual activities, where 44.3 and 81.3% of patients experienced severe problems. This observation coincides with the literature [14, 39].

The result of a recent systematic review [40] showed that HRQoL and health status are negatively associated with female gender, comorbidity, inadequate nutritional status, low physical or psychosocial functioning prior to hip fracture, longer hospital stays, and postoperative complications and pain. In our study, the univariate analysis showed correlation of the EQ-5DIndex with age, CCI, BADL, IADL, depression, pre-fracture and one-month EQ-5DVAS, type of intervention, type of fracture and functional ambulation category. We found similar results for EQ-5DVAS, although we only found six factors that correlated significantly with EQ-5D VAS. Specifically, unlike that obtained to the EQ-5D index, age, CCI, type of intervention and fracture did not correlate significantly with EQ-5DVAS, on the other hand, the EQ-5D VAS did show correlation with polymedication. The subsequent multivariate analysis confirmed the correlation between the EQ-5Dindex and the performance of basic and instrumental activities of daily living [41], and depression [42].

Consistent with what other studies have found [16, 33, 34, 43–45], we observed a correlation between higher quality of life scores in the case of hip arthroplasty versus intramedullary rod. Another factor related to a low score was institutionalisation prior to the fracture [34]. Our study was not able to refute this association; the only correlation being the one between living situation and pre-fracture index, possibly due to the low percentage of patients living in residential settings prior to the fracture. Consequently, our results suggest that patients with functional limitations prior to the fracture (low scores on Barthel Index and Lawton & Brody Scale) are at higher risk for lower HRQoL values.

We found relevant differences in the ability to perform both basic and instrumental activities of daily living. Within a month of the surgery there was a significant decrease in BADL and IADL capabilities. The greatest difference was seen in BADL, (92% of independent or mildly dependent patients prior to fracture compared to 39.3% post-fracture) because their performance requires greater physical autonomy, and because from a pre-fracture perspective only 52.7% of patients were independent or mildly dependent for IADL, which fell to 3.7%. Added to this is the fact that the IADL assessment scale analyses areas that do not require as much physical involvement, but do require the cognitive area, which in principle is not affected by the fracture or subsequent recovery, except in cases of delirium which could help to explain this difference. Hip fracture is a turning point in the patient’s living situation upon discharge from hospital with institutionalization 1 month after fracture increasing by 21.1%. In light of the results of this study, there is no doubt that the decline in functional capabilities and the need for assistance to perform daily activities makes it difficult for the patient to return to his or her family environment. Significant changes are also seen in geriatric depression assessment, with a 17.9% reduction in patients without depression. Psychosocial factors and depression symptoms may increase the severity of pain and emotional distress in patients, with direct repercussions on quality of life and, of course, functional recovery [46].

To summarise, coinciding with the findings from other studies, our study also highlights the reduction of HRQoL after hip injury, although most of these studies were conducted in other countries with cultural and health care differences and generally with longer follow-up periods. In view of these results, it is important to plan early rehabilitation programs adapted to patients’ characteristics, as well as the screening and treatment of anxiety/depression.

One limitation of the study stems from the impossibility of obtaining information prospectively on the pre-fracture situation, and therefore assuming the possibility of memory bias and underestimation of the results [47]. HRQoL in patients after hip fracture may be influenced by other unrelated factors such as pre-existing comorbidities [48]. Although we are aware of this limitation, it is intrinsically linked to studies aimed at evaluating the impact of certain pathologies on HRQoL. As with most studies, our point of reference was the pre-fracture situation 2 weeks before the fracture, on the understanding that the memory bias, if any, would be minimal since the survey is conducted at the time of admission to hospital. In addition, our multivariate analysis ruled out comorbidities (Charlson Comorbidity Index) at time of admission as an independent factor associated with HRQoL. Another limitation of the study was the impossibility of determining whether the changes identified were a direct cause of the hip fracture or were influenced by other vital situations that occurred during the study period. However, since our study looked at changes in HRQoL 1 month after hip fracture, these changes were likely the result of the fracture itself or that if there were other influences it is likely to have been in a small number of patients.

## Conclusion

Quality of life according to EQ-5D was significantly reduced after hip fracture, especially in the domains of self-care, activities of daily living, and mobility. One month after surgery, HRQoL is far from pre-fracture status. These results highlight the importance of meeting the basic needs of the elderly, not just at the time of surgery but afterwards as well, especially when problems such as pain and anxiety persist after surgery. Improved function, independence and overall quality of life are important outcomes for recovery and should be considered in rehabilitation strategies for older people following hip fracture. Further studies involving control groups, a larger cohort of patients and longer prospective follow-up are required to corroborate these results.

## Supplementary information

**Additional file 1.** Implications for practice.

## Data Availability

The datasets used and/or analyzed during the current study are available from the corresponding author on reasonable request.

## Abbreviations

ASA: American Society of Anesthesiologists
BI: Barthel Index
CCI: Charlson Comorbidity Index
EQ-5D: EuroQol-5 Dimensions 3 Levels
EQ-5D: Index
HRQoL: Health-related quality of life
*IQR*: The interquartile range
*SD*: Standard deviation
VAS: Visual Analogue Scale
