# Supplementary material for: Quality of life in elderly people after a hip fracture: a prospective study
Source: Health Qual Life Outcomes. 2020 Mar 14;18:71. doi: 10.1186/s12955-020-01314-2 (PMC7071575; doi:10.1186/s12955-020-01314-2)
Supplement: Supplementary file 1 — Additional file 1. Implications for practice. [file 12955_2020_1314_MOESM1_ESM.docx]

IMPLICATIONS FOR PRACTICE

Decision-making in the health field must take into account the perception of users, and not only traditional indicators based on clinical and economic results. This work has revealed those domains of the quality of life with greater affectation, in an early phase of the recovery after a hip fracture, as well as it has helped to determine those factors with influence in its evaluation. This information is important for the development of identification strategies and early treatment of modifiable factors that negatively affect the warm life after a hip fracture, as is the case of depression and anxiety, in addition the information provided see this work can help implement rehabilitation programs tailored to individual needs.
